# New Perspectives on Nutraceutical Insulin Sensitizing Agents in the Treatment of Psoriasis and Other Dermatological Diseases

**DOI:** 10.3390/ijms26157538

**Published:** 2025-08-04

**Authors:** Pietro Morrone, Francesca Caroppo, Alberto De Pedrini, Alessandro Colletti, Germano Baj

**Affiliations:** 1 Outpatient Specialist in Dermatology, Azienda Sanitaria Provinciale, 87100 Cosenza, Italy; 2 Unit of Dermatology, Department of Medicine, Dimed, University of Padova, 35128 Padova, Italy; 3 Regional Center of Pediatric Dermatology, Department of Women’s and Children’s Health, University of Padova, 35128 Padova, Italy; 4 Gynecology and Obstetrics, Department of Translational Medicine, University of Eastern Piedmont, 28100 Novara, Italy; 5 Department of Drug Science and Technology, University of Turin, 10124 Turin, Italy; 6 CAOM—Centro Studi Applicati Erbe Officinali e Frutti Minori, 28100 Novara, Italy

**Keywords:** insulin resistance, dermatological diseases, psoriasis, inflammation, oxidative stress, myo-inositol, vitamin D, folic acid

## Abstract

Insulin resistance (IR) plays a pivotal role in the pathogenesis of several dermatological diseases, including psoriasis, acne, acanthosis nigricans, and hidradenitis suppurativa (HS). These conditions are characterized by chronic inflammation, oxidative stress, and metabolic dysfunction, which are exacerbated by IR. This narrative review examines the emerging role of nutraceutical insulin-sensitizing agents (ISAs), including myo-inositol, alpha-lipoic acid, vitamin D, vitamin C, and folic acid, in managing IR-related dermatological disorders. A comprehensive literature search was conducted across Cochrane Library and MEDLINE (1965–May 2025), focusing on clinical trials involving nutraceutical ISAs in dermatological conditions associated with IR. Only human studies published in English were included. Evidence from randomized controlled trials (RCTs) and observational studies suggests that ISAs improve glycemic control, reduce oxidative stress, and modulate inflammatory pathways in IR-related dermatoses. Notably, myo-inositol combined with magnesium and folic acid has demonstrated significant reductions in acne severity, hirsutism, and quality-of-life impairments in women with polycystic ovary syndrome. Similar benefits have been observed in psoriasis and HS, though data remain limited. Nutraceutical ISAs offer a promising adjunctive approach for the management of IR-associated dermatological diseases, potentially addressing both metabolic dysfunction and skin inflammation. However, robust RCTs with long-term follow-up are needed to confirm these preliminary findings and to establish optimal treatment regimens.

## 1. Introduction

Identified in 1910 by Sir Edward Albert Sharpey-Shafer, insulin was initially recognized as the crucial substance missing in individuals with type 1 diabetes (T1D) [1]. It has since become one of the most groundbreaking discoveries in medical science during the 20th century, revolutionizing treatment strategies and significantly improving the life expectancy of diabetic individuals. Beyond its well-established function in glucose regulation, insulin is now acknowledged as a growth factor and a vital biological modulator that participates in various physiological processes across all tissues [2]. As scientific understanding of insulin’s metabolic role continues to grow, so does awareness of the consequences that arise from disturbances in its signaling pathways, shedding light on the pathogenic mechanisms underlying numerous diseases. Insulin, which is secreted by pancreatic beta cells, plays a central role in glucose homeostasis and also influences cellular processes such as proliferation, differentiation, and apoptosis. Insulin resistance (IR) occurs when cells become less responsive to insulin, resulting in hyperinsulinemia, heightened androgen synthesis, and metabolic irregularities. Insulin resistance (IR) is known to trigger pro-inflammatory signaling cascades, including the activation of the mitogen-activated protein kinase (MAPK) pathway and phosphoinositide 3-kinase (PI3-K) cascades, which in turn disrupt skin homeostasis [3].

**Figure 1:**
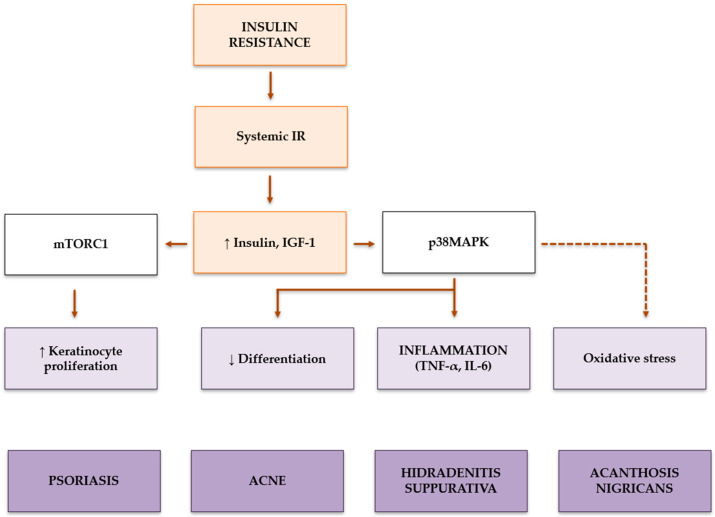
Pathogenic crosstalk between systemic insulin resistance (IR) and skin homeostasis disruption in inflammatory dermatoses. Systemic IR leads to hyperinsulinemia and elevated insulin-like growth factor-1 (IGF-1), which converge on key intracellular pathways such as mammalian target of rapamycin complex (mTORC)1 and p38 mitogen-activated protein kinases (MAPK) within the skin. Hyperactivation of mammalian target of mTORC1 fosters basal keratinocyte proliferation while repressing FoxO1, a critical transcription factor for keratinocyte differentiation and lipid synthesis, thereby impairing epidermal barrier formation. Concurrently, p38MAPK activation, fueled by pro-inflammatory cytokines like tumor necrosis factor (TNF)-α and interleukin (IL)-6, amplifies keratinocyte hyperproliferation and suppresses terminal differentiation, exacerbating cutaneous inflammation and disrupting skin integrity. These dysregulated signaling cascades are further compounded by oxidative stress and lipid dysmetabolism, creating a self-sustaining loop that underpins the pathogenesis of IR-driven dermatoses such as psoriasis, acne vulgaris, hidradenitis suppurativa. ↑ = increase; ↓ = reduction.

Several dermatological conditions, including acanthosis nigricans, acne, psoriasis, hidradenitis suppurativa, androgenetic alopecia, and hirsutism, have been linked to IR [4]. Under normal circumstances, insulin helps maintain a balance between keratinocyte proliferation and differentiation, both of which are crucial for the proper formation of the epidermal structure. However, in chronic inflammatory conditions like acne and psoriasis, elevated levels of pro-inflammatory cytokines activate the p38MAPK pathway, exacerbating IR by inducing serine phosphorylation of insulin receptor substrates (IRSs). This modification impairs keratinocyte differentiation while simultaneously promoting basal proliferation of these cells (Figure 1) [4].

The growing body of evidence surrounding the relationship between IR and skin disorders has significant therapeutic implications. There is increasing support for the use of insulin-sensitizing agents (ISAs) in managing a range of medical conditions now recognized as being linked to IR.

This review aims to explore the potential role of nutraceutical ISAs in the management of dermatological diseases associated with IR, including psoriasis, acne, acanthosis nigricans, and hidradenitis suppurativa. Although the connection between IR and metabolic disorders has been extensively studied, its relevance in dermatology remains underappreciated. This review adopts a translational and mechanistic framework that integrates the pathophysiological underpinnings of IR with emerging clinical evidence on the application of ISAs in dermatology. Through a dual focus on molecular mechanisms and interventional studies, the authors offer a comprehensive and up-to-date synthesis of how specific nutraceuticals, such as myo-inositol, alpha-lipoic acid, vitamin D, vitamin C, and folic acid, may modulate inflammatory and metabolic signaling cascades implicated in cutaneous disorders.

## 2. Overview of Insulin Signaling and Resistance Mechanisms

By regulating glucose uptake and gluconeogenesis, insulin plays a crucial role in glucose homeostasis through a complex and systemic signaling. To enhance blood glucose supply to target organs, insulin promotes the release of nitric oxide (NO) and endothelin in the endothelium, leading to vasodilation and vasoconstriction, respectively [5]. Furthermore, insulin reduces the expression of gluconeogenic enzymes in hepatocytes and boosts glycogen synthesis and enhances glucose uptake through the glucose transporter type 4 (GLUT-4) receptor in adipocytes and skeletal myocytes [6]. Moreover, insulin suppresses orexigenic neuropeptides, i.e., neuropeptide Y and agouti-related peptide in the hypothalamus, while it increases anorexigenic NP such as cocaine- and amphetamine-regulated transcript and proopiomelanocorticotropin in the arcuate nucleus [7]; this increases the activity of the α-Melanocyte-Stimulating Hormone in the paraventricular nucleus to decrease food intake [8]. By binding to its cell membrane receptor, insulin triggers autophosphorylation of intracellular tyrosine residues, resulting in the recruitment and phosphorylation of IRSs, i.e., IRS1 and IRS2 [6]. Two main intracellular pathways are described and simplified as follows:

✓ The phosphoinositide 3-kinase (PI3K) pathway, also known as the protein kinase B (PKB or Akt) pathway, facilitates GLUT-4 translocation to the membrane, glucose transport into the cell, and glycogen synthesis. This brings about the activation of PI3K by converting phosphatidyl inositol 4,5-biphosphate (PIP2) into phosphatidyl inositol 3,4,5-triphosphate (PIP3), the recruitment of Akt to the plasma membrane, and its phosphorylation by 3-phosphoinositide-dependent kinase-1 (PDK-1) and the mammalian target of rapamycin complex 2 (mTORC-2) [9]. This pathway results in the translocation of GLUT-4 in adipocytes and skeletal myocytes and the activation of glycogen synthase (GS) in skeletal myocytes, adipocytes, hepatocytes, and endothelial NO synthase (NOS) in endothelial cells, along with the phosphorylation of transcription factor forkhead box protein O1 (FoxO1) to suppress hepatic gluconeogenesis [9].
✓ The MAPK pathway, also known as the extracellular signal regulated kinase (ERK) pathway, controls transcription factors involved in cell growth, differentiation, and proliferation, while entailing the activation of ERK and the synthesis of endothelin-1 in endothelial cells [10].

**Figure 2:**
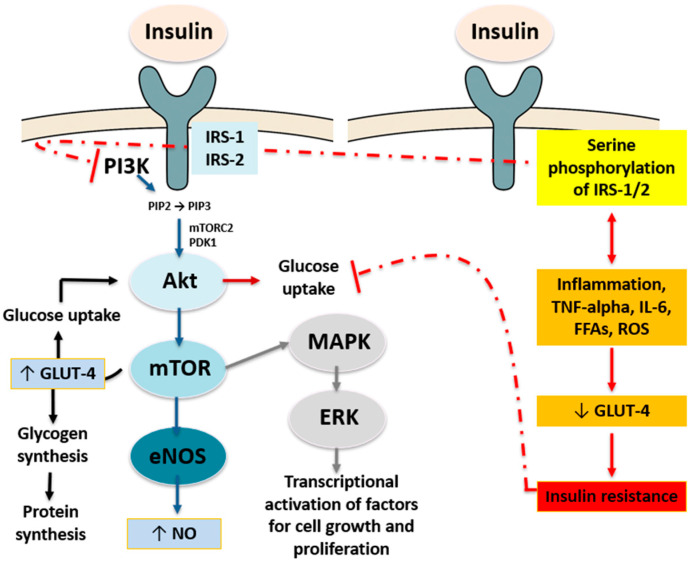
Canonical insulin signaling pathways and their impairment in insulin resistance. Upon insulin binding, the insulin receptor (IR) undergoes autophosphorylation and activates insulin receptor substrates (IRS-1/2), leading to activation of PI3K and conversion of PIP2 to PIP3. This recruits Akt and PDK1 to the membrane, where Akt is phosphorylated and fully activated by PDK1 and mTORC2. Activated Akt promotes glucose uptake via GLUT-4 translocation, glycogen synthesis, and nitric oxide (NO) production through eNOS activation. Parallel activation of the MAPK pathway regulates transcriptional programs for cell proliferation. In IR, inflammatory cytokines (e.g., TNF-α, IL-6), free fatty acids (FFAs), and reactive oxygen species (ROS) induce serine phosphorylation of IRS-1/2, impairing PI3K activation and GLUT-4 translocation, ultimately reducing glucose uptake and contributing to IR. ↑ = increase; ↓ = reduction; red dashed line = inhibition.

These molecular mechanisms (Figure 2) rely on a very delicate biochemical balance that is affected by homeostasis impairment. Inhibitory kinases which alter glucose assimilation and glycogen synthesis while stimulating gluconeogenesis by phosphorylation of IRS-1/2 are activated in the presence of inflammatory cytokines, oxidative stress (OS), and free fatty acids (FFAs) [11]. Moreover, several metabolites such as diacylglycerol and ceramides affect the modulation of insulin signaling [12]. In IR, the signaling pathways are impaired and cells do not respond appropriately to normal levels of insulin. A number of intrinsic and extrinsic factors contribute to the development of IR, including obesity, stress, and aging [13]. In the presence of IR, mitochondria are reduced in number and their morphology is disrupted with a decrease in ATP synthesis [14]. This metabolic vicious circle leads to an increase in reactive oxygen species (ROS), while greater serine phosphorylation of IRS-1/2 inhibits the insulin signaling pathway downstream, ultimately leading to an increased glucose production along with its reduced uptake, vasodilation, and insulin secretion [15]. Several additional cellular mechanisms have been recognized as potential contributors to the development of IR both in vivo and in vitro, highlighting that this condition is characterized by an overall inflamed and dysfunctional cell environment [16]. In this context, the role of biogenic amines, such as histamine, tyramine, cadaverine, and putrescine, is gaining increasing attention. These amines, produced endogenously or by gut microbiota, have been shown to modulate insulin signaling and contribute to systemic inflammation. In particular, histamine can promote pro-inflammatory cytokine release and oxidative stress, both of which impair insulin receptor function. Moreover, certain biogenic amines may influence sebaceous gland activity and hormonal balance, establishing a potential mechanistic link between insulin resistance, androgen excess, and acne vulgaris [17].

Moreover, insulin resistance (IR) is associated with decreased levels of sex hormone-binding globulin (SHBG), elevated concentrations of luteinizing hormone (LH) and follicle-stimulating hormone (FSH), and a subsequent increase in ovarian androgen synthesis, which may contribute to the development of hyperandrogenism [18,19].

IR is often described alongside the broader metabolic syndrome (MetS) that includes cardiovascular risk factors, i.e., hypertension, central obesity, impaired glucose tolerance, and dyslipidemia, which may cause non-alcoholic fatty liver disease (NAFLD) which can later progress into non-alcoholic steatohepatitis (NASH) [20]. The prevalence of IR is lowest among European adults (15.5%), while it has been reported to be as high as 23.3% in Thailand, 39.1% in Texas (US), and 46.5% in Venezuela [21,22]. Age plays a crucial role in the development of IR, since increased OS and mitochondrial dysfunction typically occur as a result of the aging process [21]. Gender must also be considered in the pathogenesis of IR as adult men normally have more visceral and hepatic adipose tissue than female subjects; this increases the presence of FFAs and pro-inflammatory cytokines that, along with the lack of the protective effect of estrogen and lower adiponectin levels, makes males more prone to IR than pre-menopausal women [21].

Besides its role in dermatoses, which is the topic of the present publication, IR plays a pivotal role in several conditions such as overactive bladder (OAB), fibromyalgia (FM), polycystic ovary syndrome (PCOS), and rheumatoid arthritis (RA), which are characterized by chronic inflammation mechanisms and not rarely associated as comorbidities by pathogenetic loops which are still not completely understood [23].

## 3. IR in Dermatological Conditions

As the largest of our organs, the skin may show signs of a serious and underlying internal disease, e.g., diabetes, lupus erythematosus, inflammatory bowel diseases, systemic sclerosis, and some types of cancer [24]. Among its many actions, insulin also affects the homeostasis and physiology of the skin as it regulates the balance between proliferation and differentiation of keratinocytes, which is a prerequisite for the formation of a healthy epidermal structure [25]. However, the highly inflamed environment which features IR leads to the activation of p38MAPK and serine phosphorylation of IRS, with a subsequent blockade of differentiation and increased proliferation of basal keratinocytes [26]. Therefore, IR is associated with pathogenesis and the severity of a few dermatological conditions which are not rarely linked to some conditions associated with overall dysmetabolism.

### 3.1. Psoriasis

Psoriasis is a systemic chronic immunomediated disorder featuring papulo-squamous plaques which consist of salmon to pink lesions covered by silvery scales, which are usually distributed symmetrically on the extensor aspects of the elbows and knees, scalp, and/or lumbosacral region [27]. However, psoriasis is an extremely complex condition which can display other skin symptoms and concomitant debilitating arthritis [28]. While obesity and overweight are considered risk and exacerbating factors for psoriasis itself, psoriatic patients are at high risk of developing metabolic diseases including type 2 diabetes (T2D), MetS, and cardiovascular conditions [29,30,31]. In particular, subjects with psoriatic arthritis (PsA) show a severe endothelial dysfunction which is directly correlated to their homeostasis model assessment (HOMA)-IR outcomes which put them at high risk of developing cardiovascular atherosclerotic comorbidities [32]. Psoriasis and metabolic diseases share an inflammatory pathogenesis, and adipose tissue (AT) is now recognized to play a role in this connection [33]. Adipocytokines such as leptin and adiponectin, which regulate and affect insulin sensitivity and signaling, are deregulated in both psoriasis and obesity, and plasma levels of adiponectin, which has anti-inflammatory action, are decreased in obesity, psoriasis, IR, and T2D [34]. Moreover, these adipokines also regulate several immune functions such as cytokine production and T-cell differentiation, emphasizing the intricate relationship between immune and metabolic dysfunctions and further supporting the link between psoriasis and IR [33]. Other adipocytokines such as omentin, visfatin, and resistin, which play an important role in insulin sensitivity, were found to be altered in psoriatics [35]. Tumor necrosis factor-α (TNF-α), a key cytokine in the pathogenesis of psoriasis, impairs insulin signaling by inhibiting the tyrosine kinase activity of the insulin receptor and reducing adiponectin secretion [36]. The association between psoriasis and IR is further supported by clinical evidence: several studies have reported significantly reduced insulin sensitivity in patients with psoriasis compared to healthy controls [37]; moreover, both serum insulin levels and IR indices positively correlate with disease severity [38]. In glucose-tolerant individuals, moderate to severe psoriasis has been linked to a marked reduction in insulin sensitivity when compared with non-psoriatic counterparts [39]. Further data have reinforced the connection between IR and psoriasis by showing a significantly higher prevalence of conditions which are normally related to IR, hyperinsulinemia, and dyslipidemia such as PCOS in psoriatic women than in healthy controls [40,41].

### 3.2. Acne

Acne is an inflammatory condition of the folliculo-pilosebaceous unit, characterized by comedones, papules, pustules, or nodules which are generally located on the face, shoulders, back, and chest [42]. Acne features a complex etiopathology which includes hyperseborrhea, hyperkeratosis, and inflammation, and it seems to be strongly associated with IR [41]. Many transcription factors such as FoxO1, 1,25-dihydroxyvitamin D and calcium are connected with sebum production, while hyperandrogenemia, hyperinsulinemia, and high levels of insulin growth factor-1 (IGF-1) play a role in acne development [43,44]. In particular, hyperinsulinemia increases keratinocytes proliferation and dysfunction by triggering IGF-1 receptors, which leads to sebocyte hyperproliferation [45]. Moreover, IGF-1 enhances androgen receptor signal transduction, elevating androgen levels and causing hyperseborrhea [46]. Increased serum insulin/IGF-1 significantly correlates with a higher dietary glycaemic load, which is a common finding in acne patients, activates mTORC1, causing IR via ribosomal protein S6 kinase b1 (S6K1), and inhibits FoxO1 which represses androgen signaling, resulting in sebaceous gland dysfunction [47,48]. Moreover, a positive correlation between a higher body mass index (BMI) and acne severity has been observed [49]. On the contrary, a lower dietary glycaemic load decreases inflammation, pro-inflammatory chemokines, and the size of sebaceous glands [24]. High glycaemia induces hyperinsulinemia and increases androgen production, while lowering the serum levels of SHBG, intensifying androgen activity, and facilitating acne development [50,51]. In patients with acne, increased mTORC1 activity has been detected, which is strongly associated with IR, obesity, T2D, and cancers such as melanoma [46]; moreover, a significant correlation between decreased expression of insulin, IGF-1, and mTORC1 and a reduced prevalence rate of acne has been observed [41]. In addition, a few studies have pointed out a direct correlation between HOMA-IR values and acne development [52].

Recent evidence also suggests that IR exacerbates oxidative stress, which plays a key role in acne pathogenesis [53]. In addition, insulin-resistant states increase ROS production in the skin, impairing keratinocyte differentiation, promoting lipid peroxidation, and activating pro-inflammatory pathways such as NF-κB and MAPK [54]. This oxidative microenvironment contributes to cutaneous inflammation and may also favor dysbiosis, increasing *Cutibacterium acnes* proliferation and aggravating acne severity [55]. These findings highlight the pathogenic synergy between metabolic dysfunction, redox imbalance, and inflammatory signaling in acne.

### 3.3. Acanthosis Nigricans

Acanthosis nigricans (AN) is characterized by velvety, hyperpigmented skin plaques typically located in areas such as the neck, axilla, and knuckles, as well as the groin, umbilical, and perianal regions [56]. Obesity-associated AN is the most common disease subtype and it is linked to IR and hyperinsulinemia [57]. Indeed, hyperinsulinemia stimulates IGF-receptors with subsequent keratinocyte proliferation [58]. The activity of IGF-1 is regulated by IGF binding proteins (IGFBPs) which increase IGF-1 half life, and IGFBP-1 and IGFBP-2 are both decreased in obese subjects with hyperinsulinemia, increasing bioactive free IGF-1 which promotes cell growth and differentiation, thereby facilitating the development of hyperkeratosis and papillomatosis observed in AN [59]. The current prevalence of AN ranges from 4.5 to 74%, and an alarming escalation of cases has been noted in younger populations affected by obesity and MetS [60,61,62]. In addition, AN has been reported as a clinical manifestation in subjects affected by Down syndrome, who are prone to obesity, MetS, and T2D [63]. In patients with AN, IR-associated skin lesions are usually located in the neck, axilla, and knuckles, even though unusual locations such as the face have been described. AN has been associated with the skin symptoms of T1D, T2D, and also PCOS [64]. Moreover, AN has been suggested as a useful clinical marker of IR particularly applicable for screening on a larger scale, such as in obese children and teenagers, populations highly susceptible to T2D and MetS, and subjects diagnosed with prediabetes status and dyslipidemia [65].

### 3.4. Hidradenitis Suppurativa

Hidradenitis suppurativa (HS) is a chronic skin disorder characterized by recurrent abscesses, draining, and scarring which involves the terminal follicular acroinfundibulum in intertriginous body areas including the armpits, the inguinal folds, the anogenital and inframammary regions, the perineum, and the nape [66]. It affects 2–4% of the population and it appears to be caused by an increased outer root sheath and keratinocyte proliferation in the follicular portion of the pilosebaceous unit with duct occlusion, rupture, and extrusion of its content, i.e., corneocytes, bacteria, yeast, sebum, and pilar residua, into the surrounding dermis with the development of a polymorphous inflammatory infiltrate [62]. Studies have reported an increased prevalence of MetS, dyslipidemia, and PCOS and higher levels of fasting plasma glucose, insulin, and tryglycerides in patients suffering from HS, while obesity is recognized as an exacerbating factor [67,68,69]. Moreover, HS is associated with a significantly increased risk of myocardial infarction, ischemic stroke, and cardiovascular disease-associated death [70]. Therefore, HS is now being recognized as a systemic inflammatory condition, even though the pathogenetic link with IR is still unclear; however, dysregulation of mTORC1 signaling and decreased adiponectin levels due to TNF-*α* overproduction have been found in patients suffering from HS [71].

## 4. ISAs in IR-Related Conditions and Dermatoses

**Figure 3:**
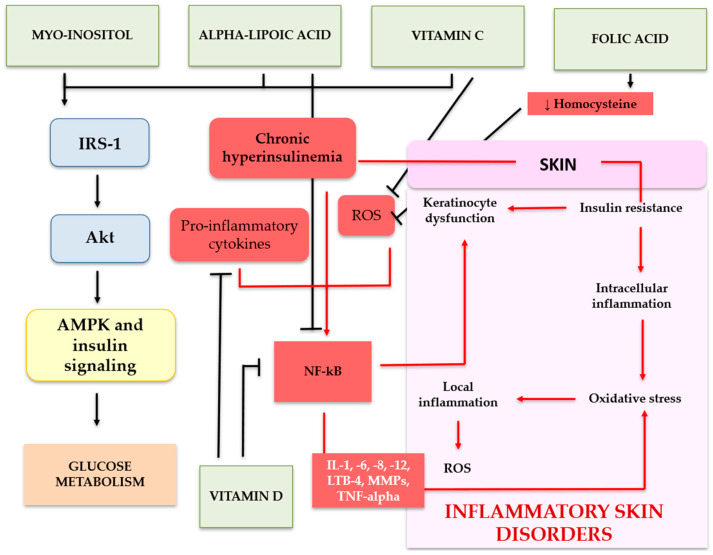
Molecular mechanisms of insulin sensitizing agents (ISAs) targeting insulin resistance, oxidative stress, and inflammation in skin disorders. The figure illustrates how nutraceutical ISAs, including myo-inositol, alpha-lipoic acid, vitamin D, vitamin C, folic acid, and B vitamins, modulate insulin signaling pathways and inflammatory responses implicated in cutaneous insulin resistance. Myo-inositol and alpha-lipoic acid enhance insulin signaling through IRS-1 and Akt, improving glucose metabolism and reducing oxidative stress via AMPK activation. Vitamin D and alpha-lipoic acid suppress NF-κB-mediated inflammation, while vitamin C supports collagen synthesis and combats reactive oxygen species (ROS). Folic acid and B vitamins contribute to endothelial function and homocysteine metabolism, indirectly alleviating systemic and cutaneous inflammation. These coordinated actions mitigate keratinocyte dysfunction, oxidative stress, and chronic inflammation, which are central to the pathogenesis of insulin resistance-associated skin diseases such as psoriasis, acne, hidradenitis suppurativa, and acanthosis nigricans.

The use of nutraceutical ISAs finds increasing application in the treatment of different diseases that share IR in their pathogenesis (Figure 3).

### 4.1. Vitamins

#### 4.1.1. Folic Acid and Group B Vitamins

Knowles et al. and Shuster et al. highlighted for the first time the possible relationship between folic acid deficiency and skin disease in patients [72,73]. Folic acid (FoA), i.e., vitamin B9/B11, plays a crucial role in red blood cell production. In trials, supplementation with FoA reduced inflammation, OS markers, and plasma concentrations of homocysteine, and it also improved glycemic control, IR, and vitamin B12 levels in T2D [74]. Further studies highlighted that FoA intake reduced HOMA-IR and improved endothelial dysfunction in individuals with MetS and that it was beneficial for glycemic markers, including Fasting Blood Glucose (FBG) and fasting insulin [75]. However, the administration of high doses of FoA may exacerbate IR [76].

In a retrospective observational study, which enrolled 98 patients suffering from chronic plaque psoriasis and 98 healthy controls, psoriasis patients exhibited higher plasma homocysteine levels compared to controls (57% of cases vs. 25% of controls; *p* < 0.0001). Plasma levels of folic acid and vitamin B12 were lower in psoriatic patients, though the difference was not statistically significant. Additionally, lower levels of vitamin B12 were observed in patients with hyperhomocysteinaemia compared to those with normal homocysteine levels (*p* = 0.0009) [77]. Similar results were also observed in people with acne. In this context, the supplementation with folic acid and vitamin B12 during isotretinoin therapy may help prevent folate deficiency and enhance blood homocysteine levels, potentially reducing the risks of cardiovascular and neuropsychiatric disorders in patients with acne undergoing isotretinoin treatment [78].

#### 4.1.2. Vitamin C

Besides its antioxidant properties, vitamin C (Vc) plays a role in lipid and glucose metabolism probably due to its ability to mediate adiponectin/adipoRII signaling [79]. Vc improves inflammatory conditions by reducing High-Sensitivity C-Reactive Protein (hs-CRP), interleukin (IL) 6, and FBG in obese patients with hypertension and/or diabetes, it is able to suppress visceral obesity and NAFLD, and it decreases hyperglycemia and blood pressure in T2D [80,81]. Vc is essential for maintaining skin integrity through several key mechanisms. First, it acts by modulating the keratinocyte differentiation and melanin synthesis, through the promotion and the differentiation of keratinocytes and inhibition of melanin production, contributing to a more uniform skin tone and offering protection against UV-induced photodamage. Second, it stimulates collagen synthesis, and it preserves skin barrier formation, which is vital for protecting against environmental insults and preventing dehydration. Third, as an antioxidant agent, Vc neutralizes reactive oxygen species generated by UV exposure, thereby mitigating oxidative stress and reducing the risk of photoaging and skin carcinogenesis [82]. Epidemiological studies showed that reduced plasma vitamin C levels have been observed in atopic dermatitis (AD) and porphyria cutanea tarda (PCT) patients, suggesting that deficiency may exacerbate the severity of these conditions [83,84]. While existing studies are promising, larger-scale, randomized controlled trials are necessary to confirm these findings and establish standardized treatment protocols.

#### 4.1.3. Vitamin D

Vitamin D (Vd), i.e., cholecalciferol or 25-hydroxyvitamin D [25(OH)D], is involved in the pathogenesis of several conditions and in the modulation of lipid profiles and inflammation [85]. Studies evidenced that supplementation with Vd improves insulin sensitivity, reduces tryglycerides, low-density lipoprotein (LDL)-cholesterol, and HOMA-IR, and is beneficial in PCOS [86,87,88,89]. Further data reported that low Vd levels are associated with a greater risk of T2D, while high levels of Vd prevent T2D occurrence [90,91]. Vd influences various skin functions, including keratinocyte differentiation, immune modulation, and barrier maintenance. In addition, it regulates both innate and adaptive immune responses in the skin [92]. In psoriasis, vitamin D deficiency is commonly observed [93,94], and supplementation with vitamin D or its analogs, such as calcipotriol, has demonstrated efficacy in improving clinical symptoms by modulating immune responses, reducing keratinocyte proliferation, and inhibiting inflammation [95]. Clinical studies have shown that vitamin D treatment reduces the severity of psoriasis, particularly in combination with other therapies like narrow-band ultraviolet B (NB-UVB) phototherapy [96]. Vitamin D also modulates the Th17 cell response, which is central to psoriasis pathogenesis, and enhances the expression of antimicrobial peptides (AMPs), crucial for skin defense [97]. In AD, vitamin D deficiency is similarly associated with disease severity [98]. Vitamin D supplementation has been shown to restore immune balance by modulating Th1/Th2 cytokines and improving the epidermal barrier, thereby reducing inflammation and enhancing skin healing [99]. Clinical trials have reported improvement in AD severity upon vitamin D supplementation, particularly with respect to skin barrier integrity and immune response [100,101]. Furthermore, vitamin D’s role in modulating AMP levels, such as LL-37, is significant in combating infections common in AD, such as *Staphylococcus aureus* [102,103].

Despite that vitamin D supplementation shows promise in managing psoriasis and AD, the effectiveness may vary due to genetic factors and environmental conditions such as sun exposure. Further studies are required to explore the exact mechanisms of action and to optimize vitamin D-based treatments for these inflammatory skin conditions and refine treatment protocols.

### 4.2. Inositol

**Figure 4:**
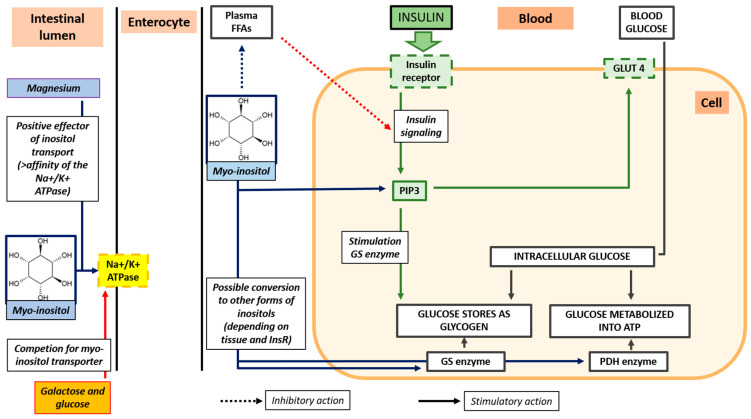
Intestinal absorption of myo-inositol, role of magnesium as positive effector of inositol transport, and myo-inositol effects on insulin resistance.

A natural isomer of glucose, inositol, is found in cell membranes as phosphatidyl-myo-inositol, the precursor of inositol triphosphate (IP3), which regulates insulin and other hormones; it is present in animal-derived food as phosphatidylinositol and in fruit and vegetables as inositol hexaphosphate (IP6) [104]. The two main inositol human isomers are D-chiro-inositol (DCI) and myo-inositol (MI) which are found in several plant-derived food, while the kidney, brain, testes, and liver can synthesize it from D-glucose at a rate of up to 4 g per day [105]. Intestinal inositol transport is driven by the sodium (Na^+^) gradient, and additional cofactors such as magnesium (Mg^2+^) increase transporter affinity (Figure 4) [106]. Pharmacokinetics investigations found that Mg^2+^ enhances the affinity of inositol transporter by 2.5 folds, suggesting that hypomagnesemia leads to reduced affinity of the inositol transport protein [107]. Other strategies used to improve inositol bioavailability include its association with α-lactalbumin, although it is a highly allergenic milk protein [108].

Inositol intake mitigates IR by improving lipid metabolism and reduces visceral fat, hepatic lipid accumulation, and insulin secretion [109]. Additionally, inositol increases adiponectin levels with a beneficial effect on adipogenesis, inflammation, and insulin sensitivity [110]. MI supplementation is effective in several conditions characterized by IR and MetS: trial outcomes on PCOS populations showed that MI enhanced gonadal parameters, improved glycemic and lipid profiles, ameliorated ovulation, oocyte maturation, and quality, and reduced HOMA-IR, hirsutism, and total androgen levels [111,112,113]; other studies reported that MI supplementation decreased the risk of gestational diabetes mellitus (GDM), macrosomia, and preterm birth, and it was also effective in diabetic nephropathy [114,115,116]. A recent systematic review assessed the efficacy of inositol, particularly MI and DCI, in treating various dermatological disorders. The review analyzed thirteen studies, including six randomized controlled trials, five non-randomized trials, one case series, and one case report, focusing on conditions such as acne vulgaris, hirsutism, seborrheic dermatitis, hidradenitis suppurativa, psoriasis, trichotillomania, and melanoma. Results showed that oral inositol supplementation demonstrated promising effectiveness in treating both PCOS-related and non-PCOS-related acne and hirsutism. Notably, no serious adverse effects were reported, highlighting its safety profile [117]. Currently, preparations containing MI and DCI in a 40:1 ratio are used for the treatment of PCOS with uncertain efficacy [118]. Based on this ratio the final product should include at least 300–1500 mg of DCI and 2–4 g of MI, while most available supplements provide much lower doses of DCI (i.e., 13.8–27.6 mg) [94]. Moreover, studies have shown that DCI may have a potentially harmful effect on oocytes [119]. In addition, DCI may act as a down-regulator of aromatase activity because it causes a possible increase in androgens and a decrease in estrogens, but the mechanism of action is still unknown [120].

Table 1 summarizes clinical studies conducted with oral MI in dermatological conditions.

### 4.3. Alpha Lipoic Acid

Alpha-lipoic acid (ALA) is a sulfur-containing fatty acid synthesized in the mitochondria, serving as a cofactor for enzymatic complexes involved in nutrient catabolism. Dietary sources include red meat, beets, carrots, potatoes, spinach, and broccoli. ALA exhibits potent antioxidant properties, neutralizing ROS through reduction of other antioxidants. Clinically, ALA is utilized in managing diabetic neuropathy by enhancing nitric oxide-mediated vasodilation and improving microcirculation [129]. It also demonstrates promise in mitigating oxidative stress-related conditions, such as ischemia–reperfusion injury and radiation-induced damage. Additionally, ALA possesses iron-chelating properties, facilitating the excretion of toxic metals like zinc, lead, and copper [130]. Being an antioxidant and an anti-inflammatory agent, ALA has been proposed recently as therapeutic option for acne vulgaris. In this regard, a preliminary study which cultured primary human sebocytes and treated them with ALA, alone or in combination with lipopolysaccharide (LPS) or dihydrotestosterone, demonstrated that ALA significantly reduced the protein expression of the inflammatory biomarkers IL-1β, IL-6, and IL-8, indicating its anti-inflammatory effect. ALA also decreased lipid peroxidation, a marker of oxidative stress [131]. In addition, ALA supplementation for age-related sensory and endothelial dysfunction in skin using two rat strains, Wistar and Brown Norway (BN), which served as models for “poorly aging” and “healthy aging”, respectively, was shown to improve vascular and sensory functions in the BN strain by the improvement of skin resistance and skin sensory thresholds and restoring the endothelial responses [132]. Therapeutic applications include ALA supplementation with dosages ranging from 600 mg to 1800 mg daily over periods up to six months. While generally safe, potential adverse effects encompass gastrointestinal disturbances, skin rashes, and hypoglycemia [133]. Contraindications involve hypersensitivity to ALA, and caution is advised in patients with thyroid disorders or those on thyroid medication. Moreover, the European Food Safety Authority (EFSA) reviewed the potential risk of insulin autoimmune syndrome (IAS) associated with the consumption of ALA. The review, based on case reports and literature reviews, concluded that ALA consumption, particularly in individuals with specific genetic polymorphisms (e.g., HLA-DRB1 * 04:03, * 04:06), increases the risk of developing IAS. This syndrome involves autoimmune-induced hypoglycemia due to insulin autoantibodies and, despite that the exact mechanism is not fully understood, it is believed that ALA may cleave insulin, altering its structure and immunogenicity, leading to antibody production. The risk of IAS was found to be relatively low in Europe but higher in countries like Japan [134].

## 5. Discussion

This review underscores the emerging role of IR as a key contributor to the pathogenesis and clinical course of several dermatological diseases, including psoriasis, acne vulgaris, acanthosis nigricans, and hidradenitis suppurativa. These conditions, traditionally managed as isolated cutaneous disorders, share common metabolic underpinnings driven by IR, systemic inflammation, and oxidative stress, aligning them within the broader spectrum of metabolic-associated inflammatory diseases [26,135]. The findings discussed herein suggest that ISAs, including MI, ALA, vitamin D, vitamin C, and FoA, may offer therapeutic benefits in managing these disorders by modulating glucose metabolism, attenuating oxidative stress, and restoring immune homeostasis. Among these, MI, particularly when combined with magnesium and FoA, appears to be the most extensively studied, with clinical trials demonstrating significant improvements in acne severity, hirsutism, and metabolic parameters in women with PCOS [122]. IR exacerbates skin conditions by altering glucose metabolism, inducing oxidative stress, and triggering chronic inflammation [136]. This disruption of cellular homeostasis contributes significantly to the pathogenesis of conditions like psoriasis, acne, and hidradenitis suppurativa. IR activates pathways that affect keratinocyte proliferation and differentiation, which are crucial for maintaining healthy skin structure. Therefore, targeting IR with ISAs offers a novel therapeutic approach for dermatological diseases previously considered to be primarily inflammatory or infectious in origin. In this context, ISAs are increasingly used in the treatment of dermatoses; for instance, metformin is recognized as an effective adjuvant agent in the therapeutic schemes of inflammatory dermatoses, endocrinology-related dermatosis, melasma, skin aging, and wound healing processes, and several trials have confirmed its beneficial use in acne, HS, and psoriasis, while semiglutide is effective in psoriatic patients with T2D [137,138,139,140,141]. Despite the effectiveness of conventional treatments for acne and other dermatological diseases, drugs are not without potential side effects. For instance, the use of metformin has been associated with gastrointestinal issues such as diarrhea, abdominal pain, and nausea [142]. These adverse effects can significantly reduce patient compliance and increase the likelihood of treatment failure. Additionally, long-term metformin use may lead to vitamin B12 deficiency, potentially resulting in hyperhomocysteinemia, a condition highly prevalent in dysmetabolic women [143]. In this context, preliminary data indicate that the use of nutraceutical ISAs, particularly MI, in treating IR-related skin disorders, such as acne and psoriasis, has shown positive results. MI’s ability to improve lipid metabolism, reduce visceral fat, and enhance insulin sensitivity is well-documented, particularly in conditions like PCOS [144]. Moreover, the combination of MI with other ISAs, like magnesium and folic acid, may enhance therapeutic outcomes. In this regard, the retrospective study by Pezza et al. evaluated the effects of a nutraceutical formulation containing myo-inositol, microlipodispersed magnesium, and folic acid on acne and hirsutism in women with PCOS. A total of 200 patients with acne and/or hirsutism participated and were treated for six months. The results indicated significant reductions in circulating androgen levels, including testosterone and dehydroepiandrosterone sulfate (DHEAS), along with improved insulin sensitivity, as evidenced by changes in basal insulin levels and the HOMA index. Acne severity, assessed by the global acne severity scale, and the impact on quality of life, measured by the Cardiff acne disability index and dermatology life quality index, improved substantially after three and six months of treatment. Furthermore, hirsutism, evaluated by the Ferriman–Gallwey score, also showed significant improvement. Importantly, no adverse effects were reported, ensuring high compliance [122]. Furthermore, an oral supplementation based on 2000 mg of MI, 56.25 mg of microlipodispersed Mg^2+^, and FoA proved to be effective in a cohort of 20 patients affected by HS, stressing the importance of an accurate assessment of metabolic profile and parameters in subjects affected by these dermatoses [128].

Several points could be addressed before ISAs can be widely implemented in clinical dermatology. First, while the agents discussed in this review are generally considered safe, there is insufficient data on their long-term safety and potential interactions with other medications commonly used in dermatology. In this regard, the effects of ISAs may vary depending on the individual’s underlying metabolic status, the severity of the dermatological condition, and genetic factors that influence insulin sensitivity. A notable limitation of the current studies is the lack of large-scale randomized controlled trials, which are essential to provide high-quality evidence on the efficacy of ISAs in managing dermatological conditions. Furthermore, the mechanisms through which ISAs exert their beneficial effects on skin health need to be better understood. While it is clear that these agents reduce inflammation and oxidative stress, the specific molecular pathways involved remain unclear.

In addition to isolated nutraceutical compounds, several botanical extracts with insulin-sensitizing properties have shown promising effects in preclinical and early clinical studies on IR-associated dermatological conditions [145]. Extracts from *Berberis aristata*, *Cinnamomum cassia*, *Galega officinalis*, *Camellia sinensis*, and *Trigonella foenum-graecum* have been reported to improve glycemic control, reduce systemic inflammation, and modulate adipokine levels [146,147,148]. Berberine from *Berberis aristata* has demonstrated the ability to activate AMPK pathways and reduce IR in both metabolic and dermatological models [149], while cinnamaldehyde from *Cinnamomum cassia* may influence GLUT-4 translocation and TNF-α expression [150]. Despite these encouraging findings, the clinical translation of botanical agents remains limited by significant challenges, including poor standardization of active constituents, batch-to-batch variability, and limited pharmacokinetic data [151,152]. Furthermore, the absence of regulatory harmonization and quality control among commercially available formulations complicates reproducibility and clinical applicability. Therefore, although plant-derived compounds represent a rich source of bioactive molecules with potential ISA activity, its integration into evidence-based dermatological care requires rigorous standardization, clinical validation, and toxicological assessment.

Detailed pharmacodynamic studies could provide insights into how these agents influence skin biology at a cellular level. More controlled studies are also required to determine the optimal dosages and treatment regimens for these agents in managing IR-associated dermatological diseases.

Bridging the gap between preclinical evidence and clinical implementation requires targeted efforts to address key translational challenges. Future studies should focus on translational strategies that facilitate the clinical adoption of nutraceutical ISAs in dermatology. First, biomarker-driven trials are warranted to identify subgroups of patients with insulin resistance-related dermatoses who may benefit most from ISA therapy, such as individuals with elevated HOMA-IR or altered adipokine profiles. Second, head-to-head comparisons between ISAs and conventional pharmacological agents (e.g., metformin, isotretinoin, biologics) are needed to evaluate relative efficacy, safety, and patient adherence. Third, formulation science and delivery systems (e.g., liposomal or micellar vehicles) should be explored to enhance ISA bioavailability and target skin tissues more effectively. Finally, a transdisciplinary approach involving dermatologists, endocrinologists, and clinical nutritionists will be essential for integrating ISA-based interventions into comprehensive therapeutic algorithms. The development of clinical practice guidelines incorporating nutraceuticals, once robust data from randomized controlled trials are available, will represent a key milestone in the evidence-based integration of nutraceutical ISAs into routine dermatological care.

## 6. Conclusions

IR is now increasingly recognized as a pathogenetic factor of several diseases as it triggers and worsens inflammation, OS, and metabolic disruption. ISAs such as MI act as metabolic regulators, antioxidants, and anti-inflammatory agents, and they are possibly effective in treating several skin disorders either when used as single agents or in association with the existing therapies. However, further clinical investigations to better assess outcomes of the use of these molecules for the treatment of dermatoses described herein are necessary.

## Figures and Tables

**Table 1:** Clinical studies conducted with oral MI in dermatological diseases.

Author(s), Year	Study Design	Participants	Intervention	Primary Outcome	Adverse Events	Follow-Up (Weeks)
Pezza et al., 2015 [121]	RCT	50 women with PCOS and acne	LEVIGON^™^ MI 2 g, microlipodispersed magnesium 56.25 mg, folic acid 200 mcg BID	Decreased number of papulopustular lesions	None	6
Pezza et al., 2025 [122]	Retrospective study	200 women with PCOS and acne	LEVIGON^™^ MI 2 g, microlipodispersed magnesium 56.25 mg, folic acid 200 mcg BID	Reduction in BMI, testosterone, free testosterone, and DHEAS levels, Improvement of quality of life (Cardiff Acne Disability Index and Dermatology Life Quality Index) and Ferriman–Gallwey score	None	24
Fruzzetti et al., 2017 [123]	RCT	50 women with PCOS	MI 4 g, folic acid 400 mcg	Reduction in BMI, 20% felt improvement in hirsutism; 38% in acne	None	6
Minozzi et al., 2008 [124]	Uncontrolled clinical trial	46 women with mild to moderate hirsutism	MI 2 g, BID	Hirsutism score decreased by −2.3 ± 0.9 (*p* < 0.001)	None	6
Advani et al., 2019 [125]	Retrospective trial	51 women (35 obese, 16 lean) with PCOS	Trazer F Forte, BID (inositol MI:DCI 600 mg, NAC 300 mg, Biotin 5 mg, Lycopene 5 mg, vitamin D 400IU)	Acne scores decreased significantly in obese patients and in lean patients)	None	3
Ramanan et al., 2020 [126]	Uncontrolled clinical trial	32 women with mild to moderate acne and hirsutism	Tracnil, BID (MI 2 g, folic acid 1 mg, vit D3 1000 IU)	Significant improvement in GA and hirsutism scores (*p* < 0.05)	Mild GI distress in some patients	6
Allan et al., 2004 [127]	Crossover RCT	23 patients with psoriasis	Inositol 6 g, QD	Patients on lithium: PASI scores improved (*p* > 0.05); patients not on lithium: PASI scores improved significantly (*p* = 0.015)	None	2.5
Donnarumma et al., 2020 [128]	RCT	10 patients with HS	Antibiotics + MI 2 g, liposomal magnesium, folic acid, BID	Reduction in Sartorius scores (*p* < 0.04)	None	6

Abbreviations: PCOS: polycystic ovarian syndrome; MI: myo-inositol; DCI: d-chiro-inositol; QD: once daily; BID: twice daily; PASI: psoriasis area and severity index; HS: hidradenitis suppurativa.

## Data Availability

Data sharing is not applicable to this article.
